# Surgically confirmed mesenteric avulsion following blunt abdominal trauma: a contemporary case series

**DOI:** 10.1007/s13304-026-02638-2

**Published:** 2026-04-04

**Authors:** M. Zafer Sabuncuoglu, Isa Sozen, Bilal Turan, Serdar Acar, İsa Karaca, Mert E. Irkin, A. Burak Erdogan

**Affiliations:** 1 Department of General Surgery, Faculty of Medicine, Akdeniz University, Antalya, Türkiye; 2 Department of General Surgery, Faculty of Medicine, Suleyman Demirel University, Isparta, Türkiye

**Keywords:** Blunt abdominal trauma, Mesenteric avulsion, Bucket-handle injury, Small bowel injury, Computed tomography, Damage-control surgery

## Abstract

**Background:**

To summarize the clinical, radiologic, and surgical characteristics of the rare but potentially fatal cases of mesenteric avulsion (MA) following blunt abdominal trauma, based on our single-center experience, and to highlight key implications for early diagnosis and management.

**Methods:**

Consecutive patients who underwent surgery between January 2017 and September 2025 and were intraoperatively confirmed to have MA were retrospectively reviewed. Demographic data, mechanism of injury, CT findings, involved intestinal segments, surgical procedures, intensive care and hospital stay, complications, and mortality were recorded. Results were analyzed descriptively.

**Results:**

A total of 13 patients (mean age, 44.5 years (20–83) were included. The most common mechanism of trauma was motor vehicle collision (69.2%). No patient showed direct CT evidence of MA (0%); all exhibited only nonspecific findings such as free fluid, solid-organ injury, or mesenteric hematoma (100%). Intraoperative involvement included jejunal (30.8%), ileal (38.5%), colonic (15.4%), and multisegmental (15.4%) regions. Segmental resection with primary anastomosis was performed in 69.2% of patients, stoma formation in 15.4%, and damage-control surgery (diagnostic laparotomy + packing) in 15.4%. The mean ICU stay was 3.1 days, and total hospital stay was 11.2 days. Postoperative complications occurred in 30.7% and resolved with conservative treatment. Four patients (30.7%) died, primarily due to concomitant multisystem or severe cranial/thoracic trauma.

**Conclusions:**

In MA cases, preoperative CT typically demonstrates nonspecific findings, making prospective diagnosis difficult. Maintaining a low threshold for early surgical exploration in the presence of hemodynamic instability, peritonitis, or a high index of clinical suspicion is essential to preserve bowel viability and reduce morbidity and mortality. Our study demonstrates that jejunal-ileal predominance, frequent use of resection with primary anastomosis, and the impact of associated multiple injuries are the major determinants of outcomes. Sustaining clinical vigilance and prompt surgical decision-making remain key to improving patient survival. Our findings emphasize that mesenteric avulsion remains largely a clinical and intraoperative diagnosis, and early surgical exploration should not be delayed based on negative or nonspecific CT findings.

**Supplementary Information:**

The online version contains supplementary material available at 10.1007/s13304-026-02638-2.

## Introduction

Small-bowel and mesenteric injuries occur in approximately 1–6% of all blunt abdominal trauma cases and are most often associated with multisystem injuries [1]. Among these, mesenteric avulsion represents an ischemic bowel injury resulting from separation of the mesentery from the intestinal loop, leading to devascularization of the affected segment [1, 2]. Shearing and traction forces generated during sudden deceleration preferentially act at the transition zones between fixed and mobile bowel segments (most notably around the ligament of Treitz and the ileocecal region) and may result in mesenteric tearing [1–4].

In contemporary practice, seat-belt and steering wheel or handlebar impacts represent the most common etiologic mechanisms, collectively referred to as the classic *“seat-belt injury”* pattern. However, rather than the absolute force of trauma, it is the degree of mesenteric stretching and the localized stress at anatomic attachment sites that primarily determine the severity of tearing [2, 3, 5].

Computed tomography (CT) is the first-line imaging modality in the evaluation of blunt abdominal trauma; however, the prospective diagnosis of mesenteric avulsion (MA) remains challenging. Reported sensitivities for CT range between 45 and 75%. The most frequently described findings include free intraperitoneal fluid, mesenteric hematoma, bowel wall hypoperfusion, and associated traumatic abdominal wall hernia. Nevertheless, these features are non-specific, and diagnosis is established intraoperatively in a substantial proportion of patients. Delays in recognition and intervention markedly increase the risk of bowel gangrene, perforation, sepsis, and mortality [5–8].

In the literature, reports of MA are exceptionally rare, typically limited to single-case descriptions or small case series [2]. To date, there is no standardized algorithm or clinical guideline addressing its diagnostic and therapeutic approach. Because the clinical presentation is often subtle and radiologic findings are frequently nonspecific, diagnosis largely depends on the surgeon’s clinical suspicion and vigilant monitoring. Therefore, detailed reporting of individual cases plays a crucial role in clarifying early diagnostic indicators and guiding surgical decision-making.

Accordingly, this study aimed to evaluate the demographic, clinical, radiologic, and surgical characteristics of 13 patients who underwent surgery for blunt abdominal trauma and were intraoperatively diagnosed with mesenteric avulsion at the Department of General Surgery, Süleyman Demirel University Faculty of Medicine, between 2017 and 2025. By comparing our findings with the existing literature, we sought to shed light on the diagnostic and therapeutic course of this rare and potentially life-threatening injury. Despite advances in trauma imaging, there is still no reliable preoperative diagnostic pathway for mesenteric avulsion, and evidence is limited to small case series.

## Materials and methods

### Patient selection

This study included patients who underwent surgical exploration for blunt abdominal trauma and were intraoperatively confirmed to have mesenteric avulsion between January 1, 2017, and September 1, 2025.

Patients were eligible if they had undergone laparotomy following trauma, if mesenteric avulsion or bowel devascularization was directly observed during surgery, and if complete preoperative imaging and postoperative follow-up data were available in their medical records.

Patients were excluded in the presence of penetrating abdominal trauma, isolated serosal injury or minor mesenteric contusion, or if they had been managed conservatively without surgical exploration.

This study was not designed to evaluate all exploratory laparotomies performed for blunt abdominal trauma. Only patients in whom mesenteric avulsion was intraoperatively confirmed were included. Cases in which exploratory laparotomy was performed but no mesenteric avulsion was identified were not included in the study cohort. Based on these criteria, a total of 13 patients were included in the study.

### Data collection and evaluation

Patient data, including age, sex, mechanism of trauma, preoperative imaging findings, time to surgery, intraoperative observations, surgical procedures performed, associated injuries, length of intensive care unit and hospital stay, postoperative complications, and mortality, were retrospectively retrieved from medical records.

Preoperative CT reports were re-evaluated from the radiology archive and classified as specific, nonspecific, or negative for mesenteric avulsion.

All diagnoses were established intraoperatively during laparotomy, and surgical management was categorized as resection with primary anastomosis, stoma formation, or damage-control surgery (packing or Bogota bag).

### Ethical approval

The study was approved by the Clinical Research Ethics Committee of Suleyman Demirel University Faculty of Medicine (date: 2025-11-03, decision no: 105/46). All procedures were conducted in accordance with the principles of the Declaration of Helsinki.

### Statistical analysis

Data analysis was performed using IBM SPSS Statistics for Windows, Version 25.0 (IBM Corp., Armonk, NY, USA). Given the limited sample size, only descriptive statistics were applied. Continuous variables were expressed as mean ± standard deviation (SD) and range (minimum–maximum), while categorical variables were presented as number (n) and percentage (%). No intergroup comparisons were conducted; results were interpreted descriptively.

## Results

During the study period, 13 patients who underwent surgical exploration for blunt abdominal trauma and were intraoperatively confirmed to have mesenteric avulsion were included in the analysis (Table 1). Ages ranged from 20 to 83 years (mean, 44.5). Mechanisms of injury were motor vehicle collision in 9 patients (69.2%), work-related blunt trauma in 2, fall from height in 1, and motorcycle crash in 1.

**Table 1:** Summary of demographic, imaging, and operative features of mesenteric avulsion cases (n = 13)

Variable	n (%) or mean ± SD	Notes
Age (years)	Mean 44.5 (range 20–83)	
Mechanism of injury	9 (69.2%) motor vehicle crash 2 (15.4%) work-related blunt trauma 1 (7.7%) fall from height 1 (7.7%) motorcycle crash	
Preoperative CT findings	0 (0%) direct signs of mesenteric avulsion 13 (100%) non‑specific findings (free fluid/solid organ injury/hematoma) 0 (0%) negative	No bucket‑handle–specific sign on CT
Associated extra-abdominal injuries	9 (69%)	Thoracic (5), cranial (4), extremity (3), vertebral (2)
Time to surgery	12 (92.3%) taken directly from ER 1 (7.7%) after short ward observation	
Involved intestinal segment (intraoperative)	4 (30.8%) jejunal 5 (38.5%) ileal 2 (15.4%) colonic 2 (15.4%) multiple segments	
Performed surgical procedures	9 (69.2%) segmental bowel resection + primary anastomosis 2 (15.4%) stoma (ileostomy/loop colostomy) 2 (15.4%) diagnostic laparotomy + packing (damage control)	“Resection length” intentionally omitted per journal preference
Additional procedures	2 (15%) Bogota bag 3 (23.1%) (e.g., diaphragmatic repair, omentectomy, adhesiolysis)	
ICU stay (days)	Mean 3.1 (range 1–7)	
Total hospital stay (days)	Mean 11.2 (range 5–22)	
Postoperative complications	4 (30.7%)	All resolved conservatively
Mortality	4 (30.7%)	Attributed to multisystem trauma

### Presentation and preliminary diagnoses

All patients presented to the emergency department with abdominal pain and/or signs of acute abdomen following trauma. The most frequent preliminary labels were “blunt abdominal trauma,” “intra-abdominal hemorrhage,” and “solid-organ laceration.” Multisystem injury was common: 9 patients (69%) had extra-abdominal pathology, including thoracic injury (hemothorax, contusion, rib fractures), cranial trauma, cervical vertebral fracture, extremity fractures, or vertebral injuries.

### Preoperative imaging

No patient had prospective CT findings diagnostic of mesenteric avulsion or a bucket-handle-type tear. Imaging most often showed nonspecific features, free intraperitoneal fluid, solid-organ laceration (spleen, liver, kidney), or mesenteric hematoma, hence the definitive diagnosis in all cases was made intraoperatively.

CT examinations were interpreted as part of routine trauma assessment at the time of patient presentation. No formal retrospective re-evaluation by dedicated trauma radiologists or interobserver agreement analysis was performed for the purpose of this study.

### Timing of surgery

Twelve patients (92.3%) proceeded directly from the emergency department to the operating room; one patient required delayed laparotomy after short ward observation for evolving acute abdomen.

### Intraoperative findings

Diagnosis was established at exploration in all patients. The most common pattern was mesenteric avulsion with associated devascularization (69.2%). Involvement was jejunal in 4 patients, ileal in 5, colonic in 2, and multisegmental in 2. Concomitant solid-organ injury (spleen, liver, or kidney) was present in 5 patients.

### Operative management

All patients underwent laparotomy. Segmental bowel resection with primary anastomosis was performed in 9 cases (69.2%); stoma creation (ileostomy or loop colostomy) in 2 (15.4%); and diagnostic laparotomy with hemostasis/packing (damage-control) in 2 (15.4%). Additional procedures in three patients included diaphragm repair, omentectomy, and lysis of adhesions (bridectomy/adhesiolysis).

### Postoperative course and outcomes

All patients were monitored in the intensive care unit postoperatively. The mean ICU stay was 3.1 days, and the mean total hospital stay was 11.2 days. Overall mortality was 4/13 (30.7%): causes of death were multisystem injury with massive intraoperative hemorrhage (n = 2) and traumatic brain injury (n = 2). Of the survivors, nine were discharged after uneventful recovery. Postoperative complications included transient paralytic ileus in three patients and a high-output stoma in one; all resolved with conservative management.

**Fig. 1:**
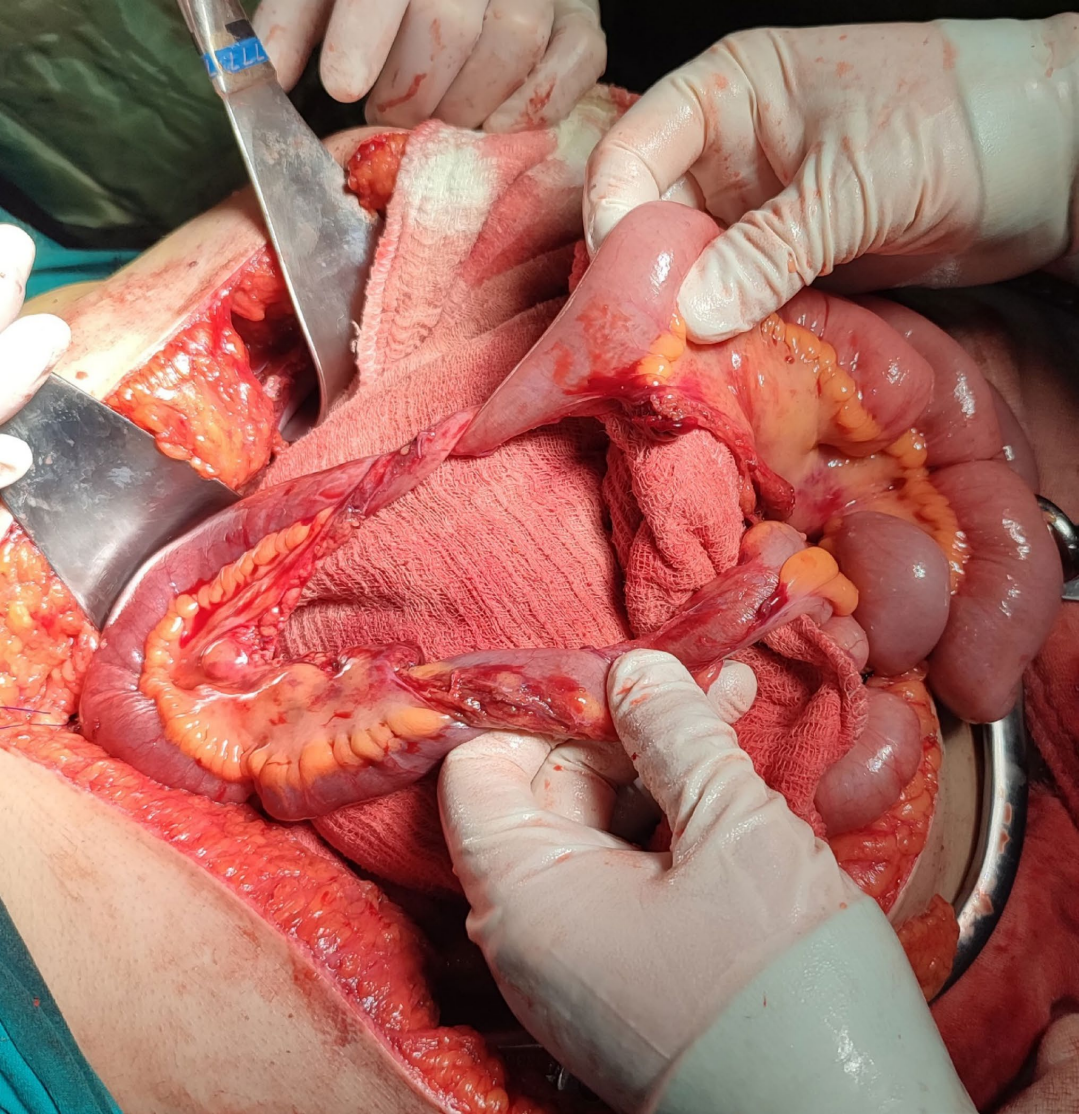
Intraoperative view of mesenteric avulsion in the jejunoileal region after blunt abdominal trauma, showing a mesenteric defect with an adjacent devascularized bowel segment

The main characteristics of the patients are summarized in Table 1. Representative intraoperative findings of mesenteric avulsion are shown in Fig. 1, with additional examples provided in Supplementary Figs. 1 and 2.

## Discussion

Mesenteric avulsion is a rare but potentially life-threatening consequence of blunt abdominal trauma. Early diagnosis remains challenging because radiologic findings are often nonspecific and clinical presentation may initially be subtle. In our series, mesenteric avulsion was not suspected preoperatively and was identified only during surgical exploration, highlighting the diagnostic challenges of this rare injury.

To provide a clearer comparison of our findings with previously published data, a summary of the main clinical series describing mesenteric avulsion and related blunt mesenteric injuries is presented in Table 2 [2, 5–7]. Most available publications consist of small and heterogeneous case series, literature reviews, or imaging-based cohorts. In contrast, our study represents a contemporary cohort of surgically confirmed mesenteric avulsion cases, allowing a detailed characterization of the involved intestinal segments and operative management strategies.

**Table 2:** Comparison of previously published series of mesenteric avulsion and bucket-handle injuries

Study	Year	n	Injury type	Design	MVC (%)	Operated (%)	Laparoscopy (%)	Laparotomy (%)	Jejunum (%)	Ileum (%)	Colon (%)	Mortality (%)
Chowdhury et al. [2]	2022	22	Bucket-handle review	Literature review	77.3	NR	NR	NR	NR	NR	NR	NR
Kordzadeh et al. [5]	2016	20	Mesenteric injury review	Systematic review	60	90	NR	90	NR	NR	NR	15
Tilden et al. [6]	2021	41	Bowel + mesenteric injury	Retrospective trauma series	NR	75.6	NR	75.6	7.3	17.1	41.5	NR
Steenburg et al. [7]	2015	126	Mesenteric injury on CT	CT imaging cohort	NR	14.3	0	14.3	NR	NR	NR	NR
Present study	2026	13	Surgically confirmed mesenteric avulsion	Surgical case series	69.2	100	0	100	30.8	38.5	15.4	30.7

*NR* Not reported in the original publication

The study by Chowdhury et al. represents a literature-based review summarizing previously reported cases of bucket-handle mesenteric injuries rather than a single-center clinical series. The Chowdhury et al. study included an additional institutional series of four patients, which was not included in this comparative analysis. Similarly, the study by Kordzadeh et al. is a literature review compiling reported cases of mesenteric and bowel injuries, rather than a primary cohort of patients managed at a single institution. The study by Tilden et al. included a broader spectrum of blunt bowel and mesenteric injuries rather than isolated mesenteric avulsion cases, which should be considered when comparing segmental distribution. The study by Steenburg et al. focused on CT imaging findings in patients with mesenteric injury rather than surgically confirmed mesenteric avulsion cases

This contemporary case series highlights the persistent diagnostic challenge of mesenteric avulsion following blunt abdominal trauma in the computed tomography era and underscores the critical role of clinical judgment and early surgical exploration. Despite systematic use of contrast-enhanced CT, none of the patients in our cohort demonstrated direct imaging findings diagnostic of mesenteric avulsion, reinforcing prior observations that radiologic evaluation alone is insufficient to reliably identify devascularizing mesenteric injuries. Importantly, our findings extend the existing literature by demonstrating that this diagnostic limitation persists even in modern trauma settings and across a spectrum of intestinal segments, including small bowel and colon. The uniformly intraoperative diagnosis in our series supports a paradigm in which timely operative decision-making, rather than reliance on imaging findings, remains central to preventing bowel ischemia and its associated morbidity and mortality in patients with suspected mesenteric avulsion.

Beyond confirming previously reported mechanisms of injury, our series provides several additional insights. First, the cohort demonstrates that mesenteric avulsion may involve not only the small bowel but also colonic segments, thereby expanding the spectrum of injury patterns previously described in the literature. Second, despite the routine use of contrast-enhanced CT in modern trauma practice, none of the patients demonstrated direct preoperative imaging evidence of mesenteric avulsion, further emphasizing the limitations of CT in detecting devascularizing mesenteric injuries. Finally, our findings highlight the continued importance of clinical judgment in trauma surgery when imaging findings are equivocal, indicating that timely operative exploration remains essential to prevent bowel ischemia.

Mesenteric and intestinal injuries resulting from blunt trauma are rare but potentially life-threatening, and delayed diagnosis substantially increases the risk of morbidity and mortality [1, 2, 9]. Because mesenteric avulsions (MAs) often present with ambiguous clinical manifestations and nonspecific CT findings, establishing the diagnosis after blunt abdominal impact is challenging and frequently requires a high index of clinical suspicion [7, 10, 11].

Kordzadeh et al. [5], in a systematic review of previously reported cases, identified motor vehicle collisions associated with seat-belt use as the most common mechanism of mesenteric avulsion injury. In line with these findings, motor vehicle crashes were also the predominant cause of injury in our cohort. Similar observations have been reported by Chowdhury et al. [2], who described seat-belt–related trauma as a frequent mechanism in their review of mesenteric injuries. Although the mean age of patients in our series was somewhat higher than that reported in earlier studies, this difference likely reflects the higher prevalence of multisystem trauma among the patients included in our cohort [5].

From a preoperative diagnostic perspective, the literature indicates that imaging findings of mesenteric avulsion are often subtle, and diagnosis is established intraoperatively in most cases [5, 11].

In our series, none of the patients demonstrated direct CT evidence of mesenteric avulsion or a bucket-handle–type tear; instead, only nonspecific findings such as free intraperitoneal fluid or solid-organ laceration were observed. This observation supports the conclusion by Kordzadeh et al., who noted that CT has low sensitivity (approximately 45–50%) for detecting such injuries [5]. Similarly, Extein et al. [1] reported that CT has limited value in identifying devascularizing mesenteric injuries, with the most common findings being mesenteric hematoma, bowel wall hypoenhancement, interloop fluid, and associated traumatic abdominal wall hernia (TAWH). In our cohort, none of these findings were prominent at presentation; thus, the diagnosis in all cases was confirmed during surgical exploration.

Patients who are hemodynamically unstable or present with signs of peritonitis or overt intra-abdominal bleeding should proceed directly to emergency laparotomy, with or without prior Focused Assessment with Sonography for Trauma (FAST) evaluation. In contrast, hemodynamically stable patients pose a greater diagnostic challenge and should undergo a contrast-enhanced CT trauma scan to identify any intra-abdominal injuries [6, 12].

Although CT is the recommended imaging modality for blunt abdominal trauma, mesenteric injuries remain difficult to detect, particularly with respect to distinguishing those amenable to conservative management from those requiring operative intervention. Bucket-handle mesenteric tears mandate urgent surgical management because of the attendant risk of devascularization followed by bowel ischemia and perforation [2].

An untreated, devascularized bucket-handle mesenteric injury can also progress to ischemia and ultimately perforation [2, 9], leading to peritonitis, sepsis, and increased morbidity and mortality. Pathophysiologically, the literature emphasizes that mesenteric avulsion separates the affected intestinal segment from its mesentery, resulting in arterial and venous devascularization; this process causes early ischemia and necrosis, and if diagnosis is delayed, may culminate in perforation [9]. In our series, devascularization frequently accompanied the avulsion intraoperatively, underscoring the importance of maintaining a low threshold for early surgical exploration.

Regarding intraoperative findings, the jejunum and ileum were the most frequently involved intestinal segments in our series. This pattern is consistent with previous studies indicating that bucket-handle-type mesenteric tears tend to occur near the transition zones of the proximal jejunum and distal ileum [1, 2]. Once surgical intervention becomes necessary, treatment options include primary repair of the mesentery, bowel resection with primary anastomosis, or resection with temporary or permanent stoma formation [10, 13]. Because the available evidence is limited and largely derived from small case series, standardized operative strategies remain poorly defined. Consequently, the choice between primary anastomosis and stoma formation often depends on intraoperative findings and the patient’s overall physiological status, particularly in hemodynamically unstable or high-risk trauma patients [10, 13].

Colonic involvement was also observed in our cohort. In cases with mesenteric avulsion or bowel necrosis, extended right hemicolectomy, ileocecectomy, or segmental sigmoid resection was required, whereas superficial serosal injuries were managed with local repair when appropriate. Overall, the operative strategies applied in our series were consistent with those reported in previous studies [2, 5], further highlighting the heterogeneous spectrum of bowel and mesenteric injuries encountered in blunt abdominal trauma.

The timing of surgical intervention in our series was also comparable to that reported in previous studies. Most patients required early operative management directly after the initial trauma evaluation, while delayed intervention was necessary only in rare cases when clinical deterioration occurred during observation. Similar patterns have been described in previous reports, where the majority of patients underwent surgical exploration within the early phase following trauma [2, 5].

Regarding postoperative outcomes, patients in our series generally demonstrated a favorable postoperative course with relatively short intensive care unit and hospital stays. Previous studies have reported variable durations of hospitalization following mesenteric avulsion injuries [1–3, 5]. The comparatively shorter recovery period observed in our cohort may be related to early surgical intervention in most patients as well as close postoperative monitoring and management.

The mortality rate in our series was 30.7%, which is higher than the 10–15% reported in the literature. Upon review of fatal cases, multisystem injuries, such as concomitant cranial and thoracic trauma, were identified in half of the patients. Similarly, Kordzadeh et al. emphasized that multiple organ involvement significantly increases mortality [5]. Nevertheless, it should be recognized that unrecognized mesenteric avulsion can also lead to bowel devascularization, ischemia, and necrosis, all of which are potentially fatal if diagnosis and intervention are delayed. Therefore, even in the absence of specific CT findings after blunt abdominal trauma, maintaining a high index of clinical suspicion, closely monitoring the clinical course, and not deferring early surgical exploration when indicated are of vital importance. Timely diagnosis and appropriate operative management remain the key determinants not only for preserving intestinal viability but also for improving overall survival.

In multitrauma patients with nonspecific or inconclusive CT findings, the decision to proceed with surgical exploration represents one of the most challenging dilemmas in trauma surgery [9, 14]. Operative exploration carries the risk of nontherapeutic laparotomy, whereas delayed intervention may lead to irreversible bowel ischemia, sepsis, and death. In such situations, clinical findings remain of paramount importance. The presence of peritoneal irritation, rebound tenderness, persistent abdominal pain, or otherwise unexplained free intraperitoneal fluid should raise suspicion for occult bowel or mesenteric injury, including mesenteric avulsion [14]. Therefore, maintaining a low threshold for surgical exploration may be justified in selected patients. In hemodynamically stable patients with equivocal imaging findings, diagnostic laparoscopy has been proposed as a minimally invasive alternative to evaluate bowel viability and detect occult injuries when adequate expertise is available in high-volume trauma centers [15]. In our series, however, operative decisions were primarily driven by clinical judgment rather than radiologic certainty, and surgical exploration ultimately proved justified in all cases.

In hemodynamically stable patients with equivocal CT findings, diagnostic laparoscopy may represent a valuable minimally invasive option for evaluating suspected bowel or mesenteric injuries and potentially avoiding non-therapeutic laparotomy [16, 17]. Recent studies have demonstrated that laparoscopy can be safely used both for diagnostic assessment and therapeutic management in selected trauma patients when adequate expertise is available [16, 18]. However, in patients with hemodynamic instability, diffuse peritonitis, or clear evidence of significant intra-abdominal bleeding, immediate exploratory laparotomy remains the standard approach.

### Limitations

This study has several limitations. First, its retrospective and single-center design inherently limits generalizability. Second, the relatively small sample size reflects the rarity of mesenteric avulsion following blunt abdominal trauma. However, this cohort represents one of the larger contemporary series with surgically confirmed diagnosis. Third, only patients who underwent surgical exploration were included, which may introduce selection bias but ensures definitive intraoperative confirmation of mesenteric avulsion. Finally, given the descriptive nature of the study and limited sample size, comparative statistical analyses were not feasible. Despite these limitations, the study provides valuable insights into the clinical presentation, diagnostic challenges, and surgical management of this rare but potentially fatal injury.

In addition, only patients with surgically confirmed mesenteric avulsion were included in this series. Exploratory laparotomies performed for suspected bowel or mesenteric injury in which no mesenteric avulsion was identified were not evaluated. Therefore, the study does not allow estimation of the incidence of negative exploratory laparotomy or the diagnostic accuracy of preoperative imaging. Furthermore, in multitrauma patients who were managed non-operatively or who were not referred for surgical exploration, the presence of mesenteric avulsion cannot be completely excluded.

Another limitation is that the exact length of bowel resection was not systematically analyzed. Operative reports did not consistently include precise measurements, and the primary focus of this study was the pattern of mesenteric avulsion and the corresponding surgical management strategies rather than the exact length of resected bowel.

## Conclusion

In conclusion, the clinical and surgical findings in our series are largely consistent with the existing literature. However, the relatively higher number of cases, the fact that all diagnoses were intraoperatively confirmed, and the absence of any preoperative CT-based diagnosis distinguish this study from previously published reports. Moreover, the detailed documentation of colonic resections and targeted bowel and mesenteric repairs in our cohort contributes to a more comprehensive understanding of the injury spectrum and operative decision-making algorithms.

Accordingly, this study provides a meaningful addition to the current body of knowledge by elucidating the true incidence, ischemic pathophysiology, clinical spectrum, and mortality determinants of mesenteric avulsion following blunt abdominal trauma. Future collaborative multicenter studies integrating surgical and radiologic data may help to better characterize the radiologic features of mesenteric avulsion and potentially support the development of standardized diagnostic algorithms aimed at earlier recognition of this rare but life-threatening injury. Clinicians should therefore maintain a high index of suspicion for mesenteric avulsion in trauma patients presenting with unexplained intra-abdominal findings, as timely surgical exploration may be crucial for preventing bowel ischemia and improving survival.

## Supplementary Information

Below is the link to the electronic supplementary material.

Supplementary file 1 (JPEG 108 kb).

Supplementary file 2 (JPEG 191 kb).

Supplementary file 3 (JPEG 140 kb).

Supplementary file 4 (JPEG 237 kb).

## Data Availability

The data that support the findings of this study are available from the corresponding author upon reasonable request.
