# Supplementary figures and images for: Surgically confirmed mesenteric avulsion following blunt abdominal trauma: a contemporary case series
Source: Updates Surg. 2026 Apr 4;78(4):1765–73. doi: 10.1007/s13304-026-02638-2 (PMC13421322; doi:10.1007/s13304-026-02638-2)

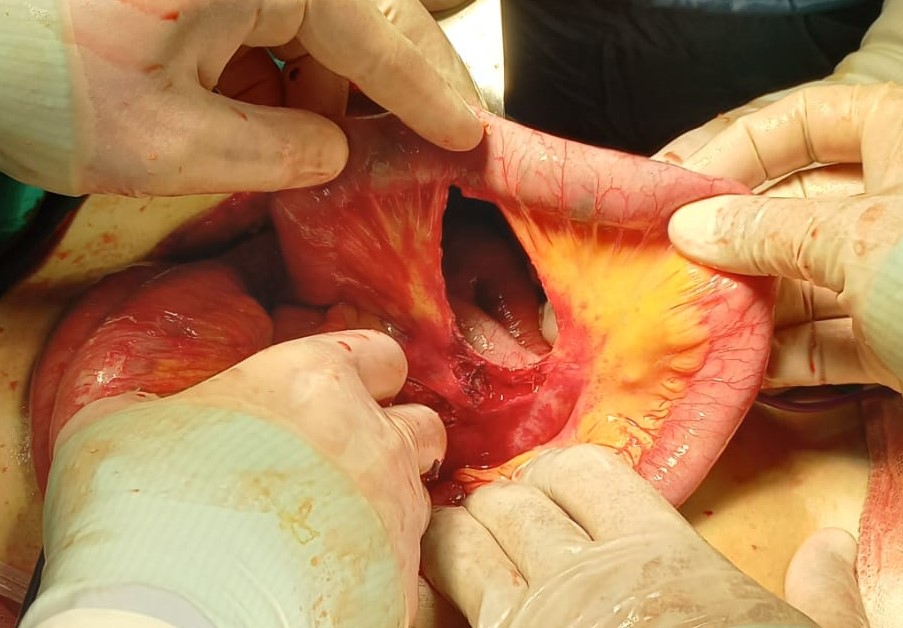

Supplement: Supplementary file 1 — (JPEG 108 kb). [file 13304_2026_2638_MOESM1_ESM.jpeg]

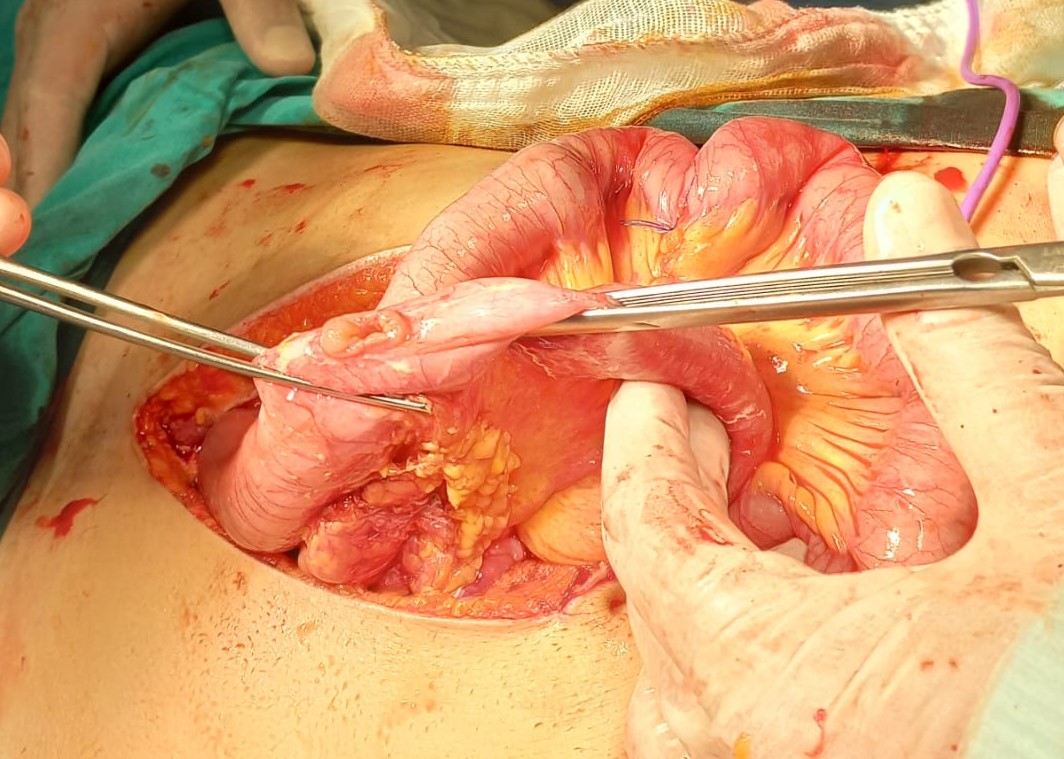

Supplement: Supplementary file 2 — (JPEG 191 kb). [file 13304_2026_2638_MOESM2_ESM.jpeg]

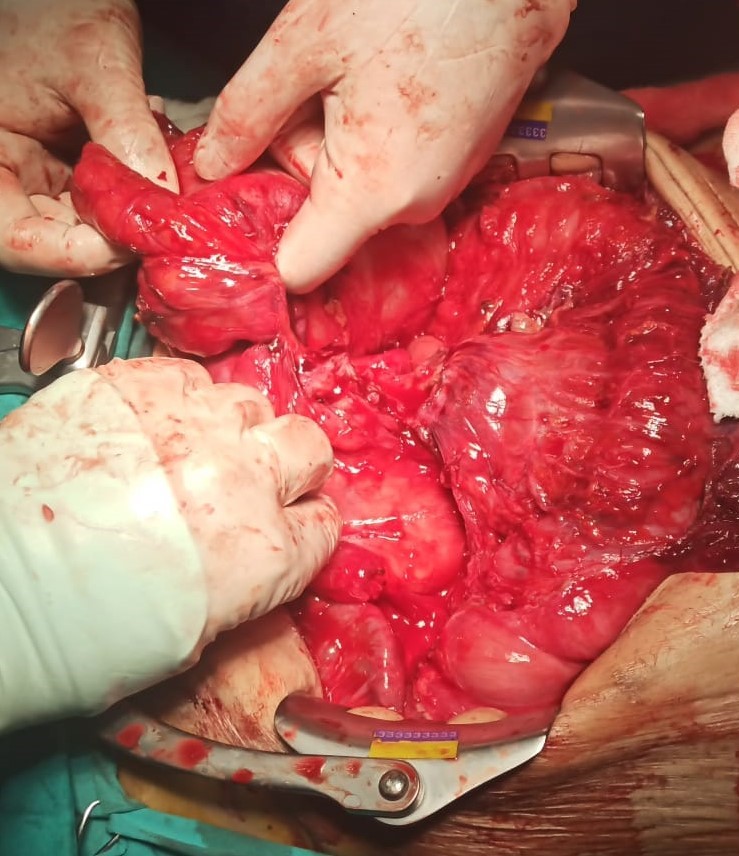

Supplement: Supplementary file 3 — (JPEG 140 kb). [file 13304_2026_2638_MOESM3_ESM.jpeg]

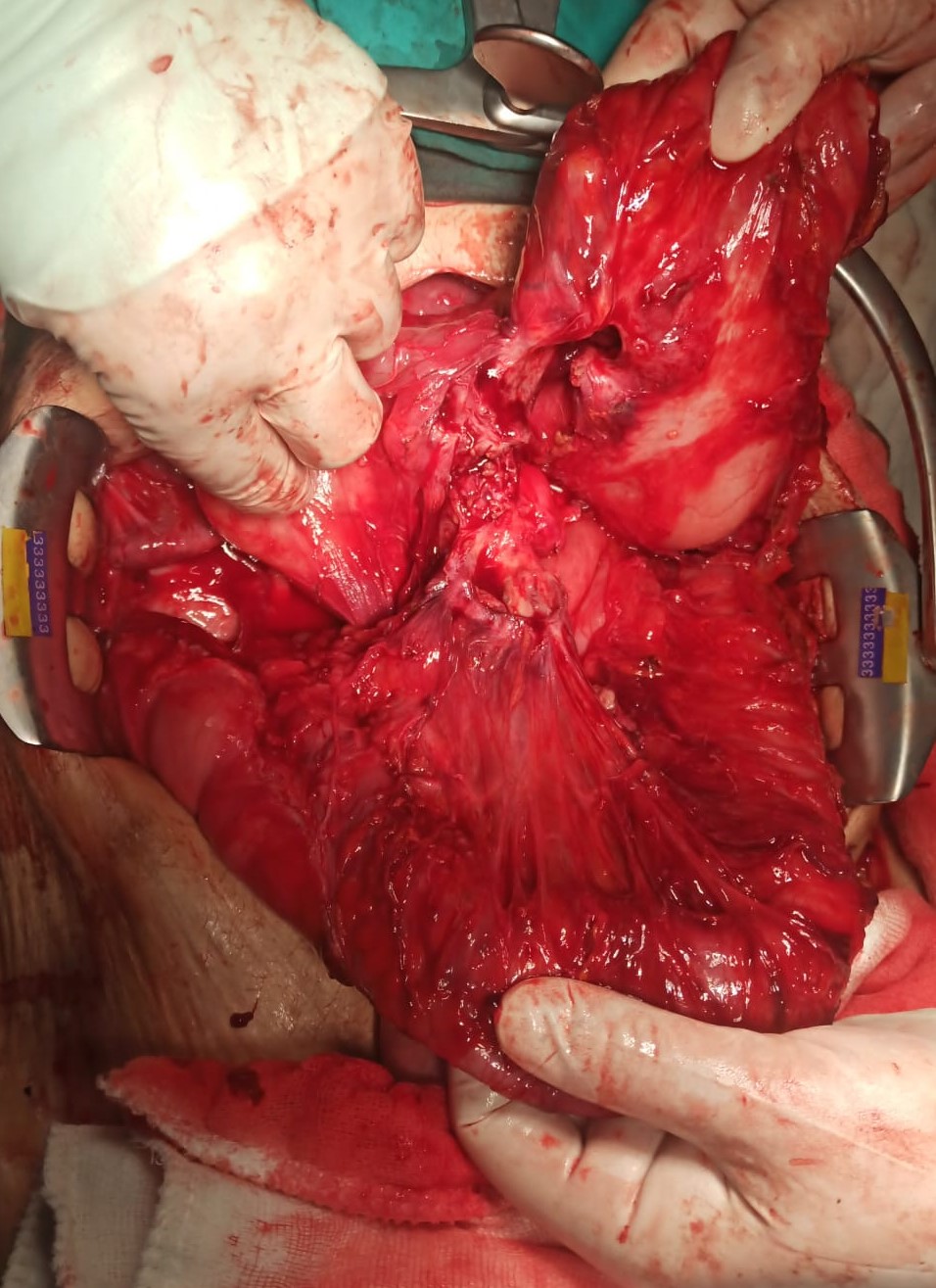

Supplement: Supplementary file 4 — (JPEG 237 kb). [file 13304_2026_2638_MOESM4_ESM.jpeg]
